# Cancer and the threat of death

**DOI:** 10.1192/j.eurpsy.2021.1979

**Published:** 2021-08-13

**Authors:** M. Regaya, B. Amamou, H. Ben Said, L. Gaha

**Affiliations:** Department Of Psychiatry, University of Monastir, Faculty of Medicine of Monastir, LR05ES10, Fattouma Bourguiba Hospital, Monastir, Tunisia

**Keywords:** cancer, death anxiety, mental health

## Abstract

**Introduction:**

Fear of death is somehow a normal sensation. Though for those having a cancer, it could increase the burden of the disease and have negative psychological impacts. This death anxiety isn’t easily verbalized by patients, thus it’s important for caregivers to manage it to improve those patients’ quality of life.

**Objectives:**

Assess death anxiety in cancer patients and to identify factors that may influence it

**Methods:**

Our study was a cross-sectional descriptive study with an analytical focus on quantitative specifications. It targeted patients hospitalized at the oncology department or consultant at the day hospital of the regional hospital of Gabes, Tunisia. Participants completed a questionnaire including sociodemographic and clinical data, using HADS scale for anxious and depressive symptoms and DAS scale for death anxiety.

**Results:**

One hundred and twenty patients were enrolled in the study. The average age of participants were 54, 9 ±11, 8 years. The majority of patients were married (68.3%) and had an average socioeconomic level (74, 2%). Our results showed that 43, 3% of patients had a high death anxiety score. Higher level of threat of death, were found in older patients(p=0.028), females (p=0.018) and for those having children (p=0,01). Death anxiety were also higher in patients having anxiety (p=0.007) and those having depression (p=0.033).

**Conclusions:**

The degree of death anxiety among cancer patients seems important. Its assessment and resolution by the caregivers remains paramount. The identification of this death anxiety should optimize the overall care of the patient.

**Disclosure:**

No significant relationships.

